# Bulimic behaviours and psychopathology in obese adolescents and in their parents

**DOI:** 10.3109/17477160903571987

**Published:** 2010-12

**Authors:** Pascale Isnard, Laure Quantin, Samuele Cortese, Bruno Falissard, Dara Musher-Eizenman, Antoine Guedeney, Marie-Laure Frelut, Marie-Christine Mouren

**Affiliations:** 1 APHP, Service de psychiatrie de l’enfant et l’adolescent, Hopital Robert DebréParis, France; 2 APHP, Service de Pédopsychiatrie, Hôpital Bichat-Claude BernardParis, France; 3 INSERM, U 669, Troubles du comportement alimentaire de l’adolescent, Faculté Paris Descartes et Paris-SudFrance; 4 Service de Psychiatrie infanto-juvénile du Dr Bentata en Seine Saint DenisFrance; 5 Service de Pédopsychiatrie, CHRU ClochevilleTours, France; 6 Service de Biostatistique, Hôpital Paul BrousseVillejuif, France; 7 Department of Psychology, Bowling Green State UniversityBowling Green, Ohio, USA; 8 APHP, Service d’endocrinologie pédiatrique, Hôpital Cochin-Saint Vincent de PaulParis, France; 9 INSERM U 675, Faculté Paris DiderotFrance

**Keywords:** Psychopathology, bulimic symptom, obesity, adolescence, parents

## Abstract

*Objective.* To help identify and advance the understanding of the potential mechanisms underlying the association between parents’ and adolescents’ psychological maladjustment in obesity, we evaluated bulimic behaviours and psychopathology in a clinical sample of obese adolescents and in their parents. *Methods.* This is a cross-sectional cohort study including 115 severely obese, treatment-seeking adolescents aged 12–17 years (mean age: 14.2; mean body mass index z-score: 4.32), and their parents (115 mothers and 96 fathers). Adolescents filled out the Bulimic Investigatory Test, Edinburgh (BITE), the Beck Depression Inventory (BDI), and the State-Trait Anxiety Inventory for Children (STAIC). Their parents completed the General Health Questionnaire (GHQ) and the BITE. A child psychiatrist filled out the Montgomery and Asberg Depression Rating Scale (MADRS) and the Brief Scale for Anxiety (BSA) for the adolescents. *Results.* Obese adolescents demonstrated significant correlations between the severity of bulimic symptoms and the degree of emotional symptomatology, such as depression and anxiety, but not with the severity of obesity. Psychopathological maladjustment and bulimic symptoms in obese adolescents were significantly associated with the maternal psychopathological disturbances, especially anxiety and somatisation in mother. In fact, maternal psychopathology, not maternal bulimic symptoms, was the factor most strongly associated with bulimic behaviours in obese adolescents. *Discussion.* These results highlight the importance of including an adolescent and parental psychiatric assessment (bulimic, depressive and anxiety symptoms), particularly maternal psychopathology in the treatment of severely obese adolescents.

## Introduction

Although the prevalence of obesity among children and adolescents in Europe and in the USA has stabilized, the rate is still high. The gene-environment interaction is a key issue in the onset and maintenance of obesity (1).

The first descriptions of the psychopathology associated with obesity in child and adolescent populations are from H. Bruch in 1940 (2): she described obesity with and without primary or secondary psychological maladjustment. Recent studies assessing the comorbid psychiatric symptoms among obese children and adolescents often show mixed results. Obese adolescents seeking clinical treatment have higher levels of depression, anxiety and eating disorders than population-based obese adolescents or than the non-obese control group (3–7). Moreover, binge eating or bulimic symptoms, frequently found in severely obese adolescents seeking treatment, are strongly related to high levels of anxiety and depression (8–12).

Although many studies show that heredity is a primary determinant of childhood obesity (13), some emphasize that the family environment may play an important role in influencing children’s eating behaviour (14). To date, few studies have investigated the parents’ weight and psychopathological factors and the extent to which they are associated with their children’s weight and psychopathological disturbances. Familial eating behaviours have indeed often been incriminated as responsible for food maladjustment and eating disorders in obese children and adolescents, and the role of parents’ influence on children’s food preferences and dietary compliance is now clearly recognized (15). Moreover, it seems that parental psychopathology (particularly maternal) but not parental weight can be associated with psychiatric problems in obese children and adolescents. Zeller et al. in 2007 (16) noted that compared with mothers of non-overweight youth, mothers of obese youth reported significantly greater psychological distress (47% of mothers of obese youth were distressed); this was not true of fathers. Epstein et al., in 1994 (17) found that parental psychiatric symptoms were related to child psychological problems assessed by their parents (particularly anxiety, depression and social problems). The relationship between mothers’ and youths’ psychological distress was confirmed by Zeller et al. in 2004 (18) using the youths’ report of their own psychological adjustment. In 1996, Epstein et al. (19) found that child’s and parental degree of obesity did not account for any variance in child psychological problems beyond that accounted for by maternal psychopathology (particularly bulimic symptoms, binge eating behaviours and depression). This is consistent with other findings, such as Vila et al. in 2004 using semi-structured interviews (20) and Decaluwé et al. in 2006 (21). This last author noted that internalizing and externalizing symptoms, as well as eating disorder symptoms in obese children were more strongly associated with mother’s psychopathology than father’s psychopathology. These results suggest that parental psychiatric symptoms are related to anxiety and depression, as well as behavioural and eating problems in obese children.

To date, however, the relationship between psychiatric symptoms in obese subjects and parental psychopathology has not been precisely evaluated to distinguish the different types of symptomatology presented by the obese subjects and by their parents, and to characterize the specific correlations between each category of problem. In addition, parents’ eating behaviours have not yet been explored to determine if and how these parental characteristics relate to the emotional and eating psychopathology of obese adolescents. These associations may be relevant both in identifying obese subjects with a higher probability of psychological distress and in understanding the potential mechanisms underlying the link between psychopathology in obese subjects and environment, especially parental contribution.

To address these gaps, this study assessed bulimic behaviours and emotional symptoms in obese adolescents and in their parents, and looked for specific relationships between these parameters. 1) We expected to find correlations between bulimic symptoms and psychopathological adjustment in obese adolescents; 2) We also hypothesized that emotional symptoms in obese adolescents would be associated with psychological symptoms in their parents (depressive and anxiety symptoms) particularly in mothers; 3) We expected there to be a correlation between bulimic symptoms in obese adolescents and in their parents; 4) Finally, we sought to confirm that adolescent and parental degree of overweight were not related to the emotional and bulimic symptoms in obese adolescents.

## Methods

### Subjects

The study sample consisted of severely obese adolescents participating in a 6 to 11 month weight loss program in an inpatient residential treatment. Criteria for inclusion in the weight loss program were: 1) seeking treatment for obesity; 2) having a body mass index (BMI) above the 97^th^ percentile; 3) being between 12 and 17 years old. Exclusion criteria for the study were: 1) symptomatic obesity from a polymalformation or a neuroendocrine syndrome, or associated with mental retardation or psychotic disorder; 2) patients or their parents unable to provide the requested information. From January 1996 to December 1999, the obese adolescents were recruited at the residential Paediatric Therapeutic Center of Margency. All were asked to participate in our research protocol: 119 families with an obese adolescent were recruited consecutively. Two families did not agree to participate in the study and two were unable to provide information because of linguistic reasons (another native language with insufficient understanding, speaking or reading French). The final study sample consisted of 115 obese adolescents and their parents (115 mothers and 96 fathers). They gave written consent to participate in the study. The assessment protocol was approved by the appropriate institutional review board from Bichat-Claude Bernard Hospital, Paris.

### Procedures and measures

A senior paediatrician (M-L.F.) from the residential Paediatric Therapeutic Center conducted a paediatric consultation before the inclusion in the weight reduction program. The paediatric assessment included a careful systematic clinical examination (measures of weight, height and stage of pubertal development) and appropriate investigations were performed in order to rule out an endocrine or genetic disorder or other organic aetiology (22). Height was measured to the nearest 0.5 cm on a standardized height board. Weight was rounded to the nearest 0.1 kg on a standard physician’s beam scale, with the subject dressed only in light underwear and no shoes. The body mass index (BMI) was calculated as weight (kilograms) divided by height (meters) squared. Subjects with a BMI above the 97^th^ percentile were included in the protocol. National BMI charts were used as the reference (23). BMI values were standardized (BMI z-scores) using age-(to the nearest 6 months) and sex-specific median, standard deviation (SD), and power of the Box-Cox transformation (LMS method), based on national norms (23). BMI z-scores were used as an index of the degree of obesity. Pubertal development was clinically assessed on the basis of Tanner stages (24).

The psychiatric assessment of the research protocol was performed at the outpatient clinic of the Department of Child and Adolescent Psychopathology of the Robert Debré Hospital, in Paris, at the beginning of the weight loss program. Demographic data (age, sex, ethnic group, socio-economic status [SES]) as well as familial and clinical history of the obese adolescents were obtained from the parents and the adolescents.

Each subject was asked to complete the following questionnaires:

1. The Bulimic Investigatory Test, Edinburgh (BITE) (25). This is a 33-item, self-report questionnaire that was designed as an objective screening test to identify subjects with bulimic symptoms. The BITE consists of two subscales: the Symptoms scale, and the Severity scale. Results from the Symptoms scale were analyzed in this study. The symptoms scale is made up of 30 items relating to symptoms (bulimic behaviours and weight control strategies), such as “Do you ever eat and eat until you are stopped by physical discomfort?”, “Can you always stop eating when you want to?”, “Do you ever eat large amounts of food rapidly (not a meal)?”, “Do you ever fast for a whole day?”. This scale has a maximum score of 30. Clinical bulimic pathology is identified by a cut-off point of 20. A score of 10 or above reflects a subclinical group of subjects who have a disordered eating pattern. The severity scale contains six items measuring the frequency by week or by day of the most significant behaviours (for example, binge eating, fasting, vomiting or diet pills taking). A score of five or above is considered to be clinically significant. The BITE is a well-validated and reliable instrument: the inter-item reliability coefficient was 0.96 for the symptom subscale and the test-retest reliability was 0.86 and 0.68 for non-clinical and clinical groups, respectively (26). This instrument was significantly correlated with other instruments measuring bulimic behaviour, such as EAT and EDI. The BITE has been used in numerous samples including adolescents all over the world: it is easy to administer and the average time for completion is less than 10 minutes (27). The BITE has been translated into French (26).
2. The Beck Depression Inventory (BDI) (28): a 13-item short form. This scale is a highly reliable and valid measure of depressive symptoms that has been used with adolescent populations. It has been translated into French and is used widely in France (29). The shortened BDI is highly related to the 21-item BDI, and was found to have a Chronbach’s alpha of 0.92, and a test-retest Spearman rho of 0.89. Each item is scored from 0 to 3 with a maximum score of 39. The cut-off score is 8 or more.
3. The State-Trait Anxiety Inventory for Children (STAIC) (30). This questionnaire measures the severity of anxiety symptoms in children. It consists of two scales, each containing 20 items. The Trait and State scales measure trait and situational anxiety, respectively. Data from the Trait subscale were used for this study. The French version has been used in several studies (31). Each item of the STAIC is scored from 1 to 3, the total score for each scale thus ranges from 20 to 60. The cut-off score for pathological anxiety in children is 34, with a sensitivity of 0.63 and a specificity of 0.75 (31). Internal consistency is 0.89 in the French version.

The parents of the subjects were asked to complete:

1. The General Health Questionnaire (GHQ) (32). This questionnaire is a detection instrument for individuals suffering from psychopathologic troubles. It contains 28 items with 4 subscales that measure somatic symptoms, anxiety and insomnia, social dysfunctioning, and depression. The GHQ has been translated into French and validated on a French population (33). The GHQ-28 is a self-administered questionnaire with scores of 0 or 1 on each item. We used the score GHQ (0-0-1-1). Higher scores reflect more severe pathological states. The cut-off score for psychopathology is 5 with a specificity of 0.91. It has good internal consistency (0.90).
2. The Bulimic Investigatory Test, Edinburgh (BITE) (25). Results from the Symptoms scale were analyzed in this study.

An experienced child psychiatrist (PI) filled out the following clinical questionnaires on depression and anxiety by using clinical interviews with adolescents:

1. The Montgomery and Asberg Depression Rating Scale (MADRS) (34). This 10-item questionnaire measures depression and is derived from the Comprehensive Psychiatric Rating Scale. Each item is scored from 0 to 6 and the cut-off score is 15. Reliability of the MADRS has been shown to be good in the literature with inter-rated reliability at 0.89–0.95, as described by Montgomery and Asberg (34). This scale is highly related to the Hamilton Depression Rating Scale (0.94). It has been translated into French and has been used in research, including studies involving adolescents (35).
2. The Brief scale for Anxiety (BSA) from Tyrer (36). This 10-item questionnaire, which assesses anxiety, is also a subscale from the English version of the Comprehensive Psychiatric Rating Scale. Each item is scored from 0 to 6 and the cut-off score is 15. It has been translated and validated on a French population (37). Although the BSA is widely used, particularly in clinical drug trials, information regarding its reliability and validity are limited but it has good concurrent validity with the Hamilton Anxiety Rating Scale and is a reliable instrument to assess anxiety (37).

### Statistical analysis

Demographic and clinical data for obese adolescents and their parents (including age, adolescents’ and parents’ BMI and adolescents’ BMI z-scores, stage of pubertal development, socio-economic status, ethnicity and scores on psychopathological questionnaires) are reported as means and standard deviation or percentages.

Categorical data were compared using the Chi-square test. Unpaired Student’s t-test analyses were used to compare means of continuous variables between boys and girls.

To assess the correlation between anxiety, depressive symptoms, bulimic behaviours, degree of obesity in adolescents and parental psychopathology and parents’ BMI, Pearson bivariate correlations were first performed considering the following variables: adolescents’ BMI z-scores, parents’ BMI, adolescents’ and parents’ BITE symptom scores, STAIC scores, BDI scores, MADRS scores, BSA scores for adolescents and GHQ scores, and the four sub-scores for parents.

To assess the independent relationship between parental psychopathology and parental eating behaviours and bulimic behaviours in adolescents, a multiple linear regression analysis was performed considering adolescents’ BITE scores as the dependent variable, and parental BITE and GHQ scores as independent variables added simultaneously.

A probability level of *p*<0.05 was used to indicate statistical significance. All statistical analyses were performed using the SPSS v.14.0 software for Windows package for personal computers (SPSS, Inc., Chicago, IL).

## Results

### Demographic and clinical data of the obese adolescents

Table I shows the demographic and anthropometric characteristics of the adolescents. Mean age was 14.2 years (±1.2) and mean BMI z-score: 4.32 (±0.78). There were significant differences between males and females with regard to age (slightly higher in girls, t=–2.84, p<0.005) and BMI z-scores were slightly higher in boys (4.69 vs. 4.15 for girls, t=3,6, p<0.001). Most of the adolescents were at stage 5 on the puberty scale and two-thirds were European. More than half of the adolescents were from low socio-economic status. Eighty-four percent of the adolescents were from married families; only sixteen percent were from single-parent families.

**Table I:** Demographic and clinical characteristics of the obese adolescents.

	Girls	Boys	All adolescents
	n=79	n=36	n=115
Age (yrs)	14.5 (1.2)**	13.8 (1.2)	14.2 (1.2)
BMI (kg/m^2^)	36.8 (6.3)	37 (5.7)	36.9 (6.1)
BMI z-scores	4.15 (0.74)***	4.69 (0.74)	4.32 (0.78)
SES			
High	19 (29.2)	10 (29.4)	29 (29.3)
Middle	10 (15.4)	6 (17.6)	16 (16.2)
Low	36 (55.4)	18 (52)	54 (54.5)
Puberty stage			
1	9 (12.2)	5 (14.7)	14 (12.2)
2	5 (6.8)	5 (14.7)	10 (8.7)
3	5 (6.8)	2 (5.9)	7 (6.1)
4	2 (2.7)	4 (11.8)	6 (5.2)
5	53 (71.6)	18 (52.9)	71 (61.7)
Ethnicity			
European	60 (75.9)	23 (63.9)	83 (72.2)
North African	7 (8.9)	6 (16.7)	13 (11.3)
Caribbean	4 (5.1)	3 (8.3)	7 (6.1)
Other	8 (10.1)	4 (11.1)	12 (10.4)

Abbreviations: BMI, Body mass index; SES, Socio-economic status. Data are shown as mean (Standard deviation) or number (percentage). Significant difference between boys and girls:

** <0.01;

*** p<0.001.

Table II reports the mean scores on the scales for obese adolescents; there were no significant differences between girls and boys. Of the adolescents, 27% were depressed on the BDI scale (BDI ≥8), 6% on the MADRS scale (MADRS ≥15), 37% were anxious according to STAIC scale (STAIC ≥34), 17% according to BSA (BSA≥15), and 6% had clinically significant bulimia according to the BITE symptoms scale (BITE above 20), and 48% had subclinical bulimia (BITE above 10).

**Table II:** Scores on psychopathological measures for obese adolescents and their parents.

	Females	Males	All adolescents
	n=79	n=36	n=115
BDI	6.3 (6.6)	7.8 (7)	6.8 (6.8)
MADRS	4.9 (4.8)	5.5 (4.6)	5.1 (4.7)
STAIC	32.8 (8.1)	32.5 (7.2)	32.7 (7.8)
BSA	8.4 (5.8)	9.1 (5.9)	8.6 (5.8)
BITE adolescent	10 (5.2)	10.9 (5.5)	10.12 (5.3)
GHQ maternal	6 (6.7)	6.4 (7.1)	6.2 (6.8)
BITE maternal	7.4 (5.8)	7.4 (6.1)	7.4 (5.9)
GHQ paternal	3.12 (5)	3.93 (5.6)	3.36 (5.17)
BITE paternal	5.1 (3.6)	5.4 (4.0)	5.2 (4.1)

Abbreviations: BMI, Body mass index; BDI, Beck Depression Inventory; MADRS, Montgomery and Asberg Depression Rating Scale; STAIC, State-Trait Anxiety Inventory for Children (Trait subscale); BSA, Brief Scale for Anxiety; BITE, Bulimic Investigatory Test, Edinburgh (Symptoms scale); GHQ, General Health Questionnaire.

Data are shown as mean (standard deviation).

### Medical and clinical characteristics of the parents

Mothers’ mean BMI was 30.67 (±7.7) and fathers’ mean BMI was 27.95 (±4.3), where 47% of the mothers and 28% of the fathers were obese (BMI ≥30 kg/m^2^).

Table II presents the clinical characteristics of the parents. There were no significant differences for parental psychopathology between girls and boys. Half the mothers and one third of the fathers had a GHQ-score ≥5 (cut-off score for significant psychopathology); thus, internalized symptoms are frequent in the obese adolescents’ parents. On the other hand, 7% of the mothers and 1% of the fathers had clinical bulimia (BITE score ≥20), and 28% of the mothers and 14% of the fathers had subclinical bulimia (BITE score above 10).

### Bulimic symptoms and psychopathology in obese adolescents

Table III shows correlation coefficients between BDI scores, MADRS scores, STAIC scores, BSA scores and BITE scores for the adolescents. In all obese adolescents, BITE index scores were significantly correlated with the psychological measures of depression and anxiety. In other words, the bulimic symptomatology increased with the severity of depression and anxiety. As expected, BDI scores, MADRS scores, STAIC scores and BSA scores were significantly correlated (p<0.0001).

**Table III:** Pearson correlation coefficients and *p* values between all obese adolescent, maternal and paternal psychopathology scores.

	BDI r (p)	MADRS r (p)	STAIC r (p)	BSA r (p)	BITE A r (p)	GHQ M r (p)	BITE M r (p)	GHQ P r (p)	BITE P r (p)
BMI z score	0.116	−0.116	−0.113	−0.086	−0.052	−0.103	−0.026	−0.214*	0.108
BDI		0.488***	0.592***	0.387***	0.514***	0.226*	0.021	0.123	0.018
MADRS		–	0.487***	0.672***	0.378***	0.307***	0.065	0.005	0.189
STAIC			–	0.456**	0.494**	0.272**	0.021	0.127	0.099
BSA				–	0.273**	0.275**	0.012	0.128	0.007
BITE A					–	0.428***	0.201*	0.111	0.13
GHQ M						–	0.43***	0.24*	0.194
BITE M							–	0.105	0.182
GHQ P								–	0.328**

Abbreviations: BDI, Beck Depression Inventory; MADRS, Montgomery and Asberg Depression Rating Scale; STAIC, State-Trait Anxiety Inventory for Children (Trait subscale); BSA, Brief Scale for Anxiety; BITE, Bulimic Investigatory Test, Edinburgh (Symptoms scale); GHQ, General Health Questionnaire; BITE A: adolescent’s BITE; GHQ and BITE M: maternal GHQ and BITE; GHQ and BITE P: paternal GHQ. Significant correlations:

* p<0.5

** p<0.01

*** p<0.001.

### Correlations between all parameters and BMI in obese adolescents and their parents

There was no correlation between the severity of obesity and bulimic symptoms or any other psychological dimensions (as shown in Table III). We did not find significant correlations between the severity of obesity in the adolescents and in their parents. Furthermore, there were no correlations between the adolescent psychological dimensions (depression, anxiety and bulimic symptoms) and the degree of parental overweight, and there was no correlation between parents* psychological symptomatology and adolescents* BMI z-scores (with the exception of a small negative correlation between the adolescents* BMI and the fathers’ GHQ scores).

### Correlations between bulimic symptoms and psychopathology in obese adolescents and their parents

Pearson correlations between the psychological parameters of the obese adolescents and their parents are also reported in Table III. Scores on the questionnaires of depression, anxiety and bulimic symptoms among the adolescents (BDI, MADRS, STAIC, BSA, and especially the BITE) were significantly correlated with maternal GHQ scores (p=0.016; p=0.002; p=0.003; p=0.005; p<0.0001, respectively). However, these scores were not correlated with maternal bulimic symptoms (BITE) except weakly with adolescent’s BITE (p=0.036). Unlike the mothers, no correlation was found between psychiatric disorders in the adolescent population and their fathers.

### Correlations with sub-score of psychopathology in the mother

As shown in Table IV, significant correlations existed between bulimic symptoms among the adolescents and all of the sub-scores of maternal GHQ (anxiety and insomnia, somatisation, depression and social dysfunction), whereas levels of anxiety and depression among the adolescents were only correlated with maternal somatisation and anxiety, and to a lesser extent with social dysfunction, but not with maternal depression.

**Table IV:** Pearson correlation coefficients and *p* values between BITE scores, MADRS scores, STAIC scores, among the obese adolescents and maternal GHQ sub-scores.

Maternal GHQ	Somatisation	Anxiety	Social dysfunction	Depression
BITE adolescent	0.479**’	0.283**	0.379***	0.303***
MADRS	0.342***	0.319**	0.202*	0.126
BDI	0.306***	0.212*	0.118	0.158
BSA	0.264**	0.309**	0.155	0.169
STAIC	0.305***	0.233*	0.203*	0.141

Abbreviations: BITE, Bulimic Investigatory Test, Edinburgh (Symptoms scale); MADRS, Montgomery and Asberg Depression Rating Scale; STAIC, State-Trait Anxiety Inventory for Children (Trait subscale); BDI, Beck Depression Inventory; BSA, Brief Scale for Anxiety; Maternal GHQ, General Health Questionnaire. Significant correlations:

* p<0.5

** p<0.01

*** p<0.001.

### Regression analysis

The multiple linear regression analysis shows that maternal psychopathology, as assessed by the GHQ scores, were significantly independently associated with the adolescents’ BITE scores (p=0.004). Table V reports the standardized beta coefficients, the standard error and the R^2^ coefficient of the regression analysis (F=3.746, p=0.007). The regression analysis indicates that 15% of the variance of the adolescent’s bulimic symptoms is explained by this model. When looking at each independent variable separately, only maternal psychopathology, as assessed by the GHQ, had a significant influence. Indeed, maternal and paternal bulimic behaviours and psychopathology of the father were not independently associated with bulimic symptoms in the obese adolescents.

**Table V:** Multiple linear regression analysis: Final model.

	R^2^	Standardized beta-coefficients	S.E.	P value
Dependent variable				
Adolescents’ BITE score	0.151			
Independent variables				
Maternal BITE		0.059	0.102	0.608
Maternal GHQ		0.344	0.091	0.004**
Paternal BITE		0.005	0.144	0.966
Paternal GHQ		0.047	0.107	0.667

Abbreviations: BITE, Bulimic Investigatory Test, Edinburgh (symptoms scale); GHQ, General Health Questionnaire; SE, standard error.

** Beta significant at *p*<0.01.

## Discussion

In accordance with previous studies (8,9,11,12), this study confirms the existence of a relationship between bulimic behaviours (for example, eating in absence of hunger, overeating, loss of control, weight control or strategy) and psychopathological symptomatology in severely obese adolescents seeking treatment. These observations underline the necessity to assess and take notice of bulimic symptoms and the comorbid psychopathology in this population: there is a subgroup of obese adolescents presenting bulimic symptoms and they are exposed to an increased risk of psychological distress that needs specific and tailored care.

The second aim of this study was to look for relationships between emotional symptoms in obese adolescents and in their parents. Our findings show that maternal but not paternal psychopathology is strongly associated with the existence of psychopathological symptoms in the obese adolescents, which is consistent with earlier studies (16,17,19,20). Our results reflect also that maternal anxiety, somatic symptoms and social dysfunction are related to the degree of emotional distress among adolescents, which is congruent with a previous study (38). In other words, the more their mothers are emotionally disturbed, the greater the severity of depression and anxiety in the obese adolescents. The relationship between parental and obese adolescent psychological characteristics remain unclear. Genetic and environmental factors may play a role and interact (38,39,40). The relationship between psychological characteristics in obese adolescent and in their mother could be explained by environmental factors: maternal psychopathology, family conflicts, or family stress factors (16,21). Another hypothesis is that maternal anxiety is partly due to disease-related stress in mothers of obese adolescents seeking treatment and suffering from psychological maladjustment (41).

The third aim of this study was to look for a relationship between bulimic symptoms in obese adolescents and in their parents. For the first time in the literature we show that there is a strong relationship between bulimic symptoms in obese adolescents and in their mothers. The same report has been mentioned by Lamertz et al. in 2005, but this was in young children (42). Thus our observations suggest that there is a link between eating behaviours in obese adolescents and their mothers probably including genetic and environmental components, as familial aggregation has been previously reported by Mangweth et al. (43). This relationship was not found between bulimic behaviour in the obese adolescent and their father, accentuating the importance of the role of maternal characteristics.

Our major finding is that bulimic behaviours in the obese adolescents were associated with all measures of maternal psychopathology, including all maternal distress (somatisation, anxiety, depression, social dysfunction). Therefore, there is also a link between psychopathology in the mother and eating disorder pathology in an obese adolescent. Parental psychopathology is also acknowledged as a risk factor for bulimia nervosa (44). This relationship could be explained first by shared genetic vulnerability between eating and mood disorders, second by the fact that depression and anxiety in the mother is perceived by the obese adolescent, which could enhance bulimic symptoms in the adolescent, and third, because bulimic behaviours in an obese adolescent could have a negative psychological impact on their mother. This explained the familial co-aggregation of eating disorders with mood disorders, as previously described (43,45,46). This relationship was not found with fathers.

In contrast, and contrary to a previously described study (47), no relationship between bulimic behaviour in the mother or father and depressive or anxiety symptoms in obese adolescents were found in our research, but the size of the sample may have been too small to find a correlation. As previously described in the literature, we confirmed that adolescent’s and parental degree of overweight were not related to emotional and bulimic symptoms in obese adolescents (19.–21).

Concerning socio-economic status, our sample was not strictly representative of the French population, the percentage of low socio-economic status was slightly higher than in the general population, as is usually described in studies involving obese adolescents, but the percentage of high socio-economic status was also higher, probably because of recruitment bias in a hospital linked to a university.

In conclusion, this study underscores the importance of maternal psychological factors in understanding severe obesity in adolescents, with the major finding that a strong relationship exists between maternal emotional symptoms and bulimic symptoms in their adolescent with severe obesity. This relationship could function in two directions: a mother with anxious or depressive symptoms could lead obese adolescents to have disordered eating behaviours, such as bulimic symptoms, or obese adolescents with bulimic symptoms could induce anxiety and depression in their mother; finally they could also share common genetic vulnerability. On the other hand, the psychological symptomatology and eating behaviours of fathers play a less important role.

Several factors increase the strength of our results: the use of a standardized procedure (BMI z-scores) to assess the level of overweight; the use of well-validated psychological instruments (except perhaps for BSA, although this scale is widely used); the high participation rate, which minimizes sample selection bias. But some of the results from this study should be interpreted with care. Indeed, we analyzed a clinical sample of severely obese patients seeking treatment in a specific department, which can elicit a concentration of more complicated pathologies, including greater psychological distress and more eating disorders than obese subjects who do not seek treatment. So our conclusions may not be extended to the general (non-clinic) population of obese adolescents and to moderately obese adolescent clinical samples. Second, parents’ psychological problems were measured only by self-report questionnaire: GHQ is filled out by the parents, which can induce a bias in the response, contrary to the adolescents’ psychopathology that was both evaluated by the adolescent and the psychiatrist. Third, we could not explore the presence or absence of disorders on the basis of the questionnaires, but only psychological dimensions. An additional limitation is that the cross-sectional design does not allow for making predictions and longitudinal research in this area is needed.

In spite of these limitations, our study confirms the close relationship between bulimic behaviours and emotional disorders among severely obese adolescents seeking treatment for their obesity. Moreover, our results reveal the importance of parental psychopathology, in particular maternal internalizing problems, in the comorbid eating and emotional maladjustment of the obese adolescents. Based on the present findings, we suggest a more direct focus on the mother’s psychological characteristics and family-based treatment, or a need for emotional support for mothers to perhaps improve the care we provide for the severely obese adolescents, leading to better progress, quality of life and social and familial functioning for both adolescents and their parents. Furthermore, larger controlled studies are necessary to confirm these results and future research should investigate the mechanisms through which parental characteristics, particularly maternal psychopathology, is linked to the obese adolescents’ eating and emotional disorders, and the influence of maternal psychopathology and emotional support for mothers in the treatment outcome of obese adolescents. This confirms the necessity of a multidisciplinary assessment and treatment of severely obese adolescents, including parental evaluation and particularly maternal psychological symptoms. More research in this domain will clarify knowledge of the mechanisms involved and may help us to modify dysfunctional associations, which in turn can improve the treatment and the adjustment of the adolescent.
